# Identification of Factors Driving Doxorubicin-Resistant Ewing Tumor Cells to Survival

**DOI:** 10.3390/cancers14225498

**Published:** 2022-11-09

**Authors:** Semyon Yakushov, Maxim Menyailo, Evgeny Denisov, Irina Karlina, Viktoria Zainullina, Kirill Kirgizov, Olga Romantsova, Peter Timashev, Ilya Ulasov

**Affiliations:** 1 Group of Experimental Biotherapy and Diagnostics, Institute for Regenerative Medicine, World-Class Research Centre “Digital Biodesign and Personalized Healthcare”, I.M. Sechenov First Moscow State Medical University, 119991 Moscow, Russia; 2 Laboratory of Cancer Progression Biology, Cancer Research Institute, Tomsk National Research Medical Center, Russian Academy of Sciences, 634009 Tomsk, Russia; 3 Research Institute of Pediatric Oncology and Hematology at N.N. Blokhin National Medical Research Center of Oncology, Ministry of Health of Russia, 115478 Moscow, Russia; 4 World-Class Research Centre “Digital Biodesign and Personalized Healthcare”, Sechenov First Moscow State Medical University, 119991 Moscow, Russia

**Keywords:** Ewing sarcoma, scRNA-seq, proteomics, doxorubicin, cell resistances

## Abstract

**Simple Summary:**

It is known that doxorubicin is one of the standards for chemotherapy treatment against Ewing sarcoma. Despite its widespread use, doxorubicin treatment initiates tumor escape mechanisms and disease relapse. Our study aims to identify the potential biomarkers of doxorubicin resistance in primary cultures of Ewing sarcoma cells using single-cell transcriptomic and proteomic analyses. To assess the specificity of identified gene biomarkers, we used publicly available datasets to represent mRNA profiles of patient samples and short-lived cultures of tumor cells, established earlier. Through our investigation, we confirmed that MGST1 and the new marker COL6A2 are both produced by doxorubicin-resistant cells and demonstrated clinical significance for the survival of patients with Ewing sarcoma.

**Abstract:**

Background: Ewing sarcoma (ES) cells exhibit extreme plasticity that contributes to the cell’s survival and recurrence. Although multiple studies reveal various signaling pathways mediated by the EWSR1/FLI1 fusion, the specific transcriptional control of tumor cell resistance to doxorubicin is unknown. Understanding the molecular hubs that contribute to this behavior provides a new perspective on valuable therapeutic options against tumor cells. Methods: Single-cell RNA sequencing and LC-MS/MS-based quantitative proteomics were used. Results: A goal of this study was to identify protein hubs that would help elucidate tumor resistance which prompted ES to relapse or metastasize. Several differentially expressed genes and proteins, including adhesion, cytoskeletal, and signaling molecules, were observed between embryonic fibroblasts and control and doxorubicin-treated tumor cell lines. While several cancer-associated genes/proteins exhibited similar expression across fibroblasts and non-treated cells, upregulation of some proteins belonging to metabolic, stress response, and growth pathway activation was uniquely observed in doxorubicin-treated sarcoma cells, respectively. The novel information on differentially expressed genes/proteins provides insights into the biology of ES cells, which could help elucidate mechanisms of their recurrence. Conclusions: Collectively, our results identify a novel role of cellular proteins in contributing to tumor cell resistance and escape from doxorubicin therapy and contributing to ES progression.

## 1. Background

Ewing sarcoma (ES) is a primary tumor that affects bones [1]—and much less often, soft tissues [2]—with a greater incidence in young people aged 10–15 years. The survival rate for ES patients has improved significantly with combined treatment, including surgery, chemotherapy, and radiation; however, this harsh treatment leaves patients with disabilities. Additionally, most of the time this disease occurs locally, with 5-year event-free survival up to 78% [3], but almost 20% of patients will experience localized disease relapse and later produce metastases (with an overall survival rate of around 28% [4,5]). Although the lung is the most prevalent location for ES metastases (85% of all patients), a recent investigation suggests that bone metastases become a valuable predictor for lung metastases during ES progression [6]. Therefore, understanding the mechanisms of tumor escape from therapy will lead to the development of better therapeutic options that are needed to elevate the therapeutic effect of current modalities and improve the outcome.

Progression and metastasis of ES is a complex biological process that involves bone lytic destruction and tumor cell propagation. It has been demonstrated that ES cells initiate osteolysis on the border between normal and neoplastic tissue via increased immobilization of macrophages, since they have been involved in the destruction of the bone extracellular collagen-containing matrix (ECCM) [7]. Along with the activation of osteoclast differentiation [8], ECCM destruction lays a foundation for tumor vascularization in the area of bone resorption and activation of angiogenesis [9]. The last step is critical for tumor cell spread and dissemination to the nearby tissue. The development of distant metastases requires the tumor cells to have an elevated level of matrix-cell adhesion proteins that allow tumor cells to attach and penetrate tissue. Several studies, particularly in the last decade, hypothesized that proteins of the carbonic anhydrase group, more specifically CA9 [10] and cadherin-11 [11], act as regulators of processes associated with sarcoma migration, invasion, and metastasis. Although recent studies provided further evidence for these [12] and other [13] proteins, such as MGST1 [14], to be involved in tumor dissemination, these proteins appeared to share an ability to regulate the formation of metastases in various cancers [15]. Despite scientific reports that various chemotherapeutic drugs [16,17] induce prometastatic proteins, therefore the tumor escape molecular mechanisms are not fully understood and have limited therapeutic efficacies against ES. 

To understand the processes that are responsible for tumor cell survival, and compare our findings with data published in the field of ES resistance to DOX [18], we aim to identify the molecular basis for the cellular reaction in response to doxorubicin stress, which precedes most ES escapes from stress and generates tumor tissue growth. In this study, we used RNA-seq analysis to assess differentially expressed genes (DEGs) using the Gene Expression Omnibus (GEO) database. We observed the upregulation of 992 genes. To validate the specificity of gene expression and their attribution to the tumor cells, we performed single-cell RNA-seq and tandem mass tag (TMT) proteomic approaches to investigate the transcriptome and proteome changes of patient-derived ES culture in comparison to embryonic fibroblasts (M19). Through transcriptomic analysis, we found 45 (27 up- and 18 downregulated) DEGs, and at the protein level, we found 124 (44 up- and 78 downregulated) differentially expressed proteins (DEPs) in doxorubicin-treated ES cells vs. doxorubicin-treated M19 cells. Furthermore, a comparison of proteomic data of doxorubicin-treated fibroblasts and doxorubicin-treated ES cells identified a unique protein signature of 11 markers, among which COL6A2, PSME1 and FLNC demonstrated clinical significance by survival analysis GSE63155 for ES patients. Our results highlight key regulatory proteins that coordinate the response of ES cells to the doxorubicin treatment by integrating signaling from MGST1 cellular metabolism, and the COL6A2 molecule, whose interactions were underappreciated in the previous investigations. This adds to our knowledge of the molecular regulatory network of doxorubicin resistance and the transcriptional hub that governs tumor escape from doxorubicin, and might hold promise as a future therapeutic target.

## 2. Materials and Methods

### 2.1. Materials

DMEM media, glutamine, fetal bovine serums (FBS) and protease inhibitor cocktail tablets (EDTA-free) were purchased from Roche Diagnostics (GmbH, Mannheim, Germany). 

### 2.2. Primary Tissue Samples

Our study included 14 tumor tissue specimens with a confirmed diagnosis of ES and treated in 2020–2021 at the clinical facility of NIMC N.N. Blokhin. All parents of sick children gave written informed consent to use their clinical data in agreement with approval by the institutional ethics committee (Research Institute of Pediatric Oncology and Hematology at N.N. Blokhin National Medical Research Center of Oncology). Based on an agreement between Sechenov First Moscow State Medical University and NIMC N.N. Blokhin, the clinical specimens were available to isolate tumor samples. Under this agreement, we established a few primary cultures from ES tumor samples; however, for the current study, we used only ES36 cells. The percentage of tumor cells in each resected tissue was more than 80% (pathologist assessment). The patient who was a donor for ES36 sarcoma cells developed a tumor mass around the tibia and later received pilot anti-relapse treatment under EE2008 protocol. However, around 1 year later this patient still relapsed.

### 2.3. Cell Cultures

Human primary Ewing sarcoma ES36 cells and human embryonic fibroblasts M19 were established as described [19]. All cell lines were routinely tested for purity and mycoplasma contamination. Human sarcoma cell line ES36 (from a 4-year-old male) and M19 (from a 9-year-old female) were grown in DMEM media, supplemented with 10% FBS and l-glutamine (1%) at 37°, 5% CO_2_. M19 cells were received from colleagues at the National Research Center for Epidemiology and Microbiology named after Honorary Academician N.F. Gamaleya of the Ministry of Health of the Russian Federation. The patient-derived ES sarcoma was used in passage 3. ES36 and M19 were authenticated using STR protocol (24 markers) at Sistema Biotech (Moscow, Russia). The multiplex analysis was conducted using the 3500 Genetic Analyzer (Applied Biosystems, Waltham, MA, USA) and data were assessed via GeneMapper ID-X v1.4 (Applied Biosystems, Waltham, MA, USA). The received profiles of cells were matched against ATCC-based STR profiles. Additionally, EWSR1/FLI1 fusion was PCR-amplified using primers, visualized with agarose gel, and sequenced. ES36 and M19 cells were used to titrate Doxorubicin (10 Mm stock), and to obtain IC50. We further selected a dose of Dox 10-fold lower than IC50 at ES36, and with no impact on M19 proliferation. Under these conditions, we incubated ES36 and M19 for 7 days with Dox(0.125 mkM) before analysis.

### 2.4. cDNA Library Preparation and Single-Cell RNA-Seq

Single-cell cDNA libraries were prepared using Single Cell 3′ Reagent Kit v3.1 and a 10× Genomics Chromium Controller. The number of cells in each channel of the Single-Cell Chip G did not exceed 5000. The dsDNA High Sensitivity kit measured the concentration of cDNA libraries on a Qubit 4.0 fluorometer (Thermo Fisher Scientific, Waltham, MA, USA). The quality of cDNA libraries was assessed using High Sensitivity D1000 ScreenTape on a 4150 TapeStation (Agilent, Santa Clara, CA, USA). The ready cDNA libraries were pooled, denatured, and sequenced on NextSeq 2000 (Illumina, San Diego, CA, USA) using pair-end reads (28 cycles for reading 1, 91 cycles for reading 2, and 8 cycles for the i7 index). Cell Ranger software (version 6.1.1) provided by 10× Genomics (version 6.1.1) (Pleasanton, CA, USA) was used to perform sample demultiplexing, alignment to the GRCh38 transcriptome, filtering, barcode counting, and UMI counting. The resulting datasets, M19 and ES36, were subjected to quality control. The subset was produced at a mark of 25% for mitochondrial material and recorded duplicates for each of the samples. Functional annotation was performed using R gProfiler2 gene ontology analysis [20].

### 2.5. Proteomic Data Generation and Analysis

We extracted proteins from the live ES36 patient-derived ES adherent cells grown in DMSO (*n* = 3) or doxorubicin (*n* = 3) using the methanol–chloroform method. Then, each sample was treated with 100 mM (NH4)_2_CO_3_ (pH 7.8) in the presence of 0.2% ProteaseMax solution (Promega, Madison, WI USA), before sonication for 10 min, followed by treatment with 100 µM Tris(2-carboxyethyl)phosphine during 1 h incubation at 55 °C and 100 µM Iodoacetamide (1 h incubation at room temperature). A digestion mix of 0.1% of trypsin and 0.2% of Protease Mix were added to each reaction for a further 3 hours of incubation and then the reaction was terminated. The protein concentration was measured using a «BCA protein assay kit» (EMD Millipore, Billerica, MA, USA) and the rest of the sample was dry-vacuumed using Centrivap (Labconco, Kansas City, MO, USA) at +45 °C. The digested and enriched peptides were dried in a speed vacuum and reconstituted with 0.1% formic acid in water for liquid chromatography-mass spectrometry (LC-MS/MS) analysis. An Q Exactive HF-X Mass Spectrometer (Thermo Fisher Scientific, Waltham, MA, USA), coupled to a Dionex 3000 RS Nano (Thermo Fisher Scientific, Waltham, MA, USA), was used to analyze digested peptides. We performed MS/MS data acquisition, converted data using an mzML format (XML (eXtensible Markup Language)-based common file format for proteomics mass spectrometric data), and processed with ThermoRawFileParser software (Thermo Fisher Scientific, Waltham, MA, USA) based on two protocols: the first used Identipy + Biosaur + Scavager to distinguish hybrid spectra. In the second we used MSFragger + Philosopher(peptideprophet/proteinprophet) + IonQuant for data quantification. Spectra were searched against species-specific TrEMBL protein databases generated from the human genome and bovine serum proteins, detected early by us in fetal bovine serum samples.

The study of relative changes in protein quantities was carried out using the DEP software package, which is based on the LIMMA statistical package (an empirical Bayesian method developed initially for the analysis of microchip data). For protein quantification, counts from peptide spectrum matches (PSM) were normalized and filtered using the MNAR (missed-not-at-random) method, log2-transformed, and hierarchically clustered by expression patterns through the time series in R., using the entire protein-coding gene set as a background.

### 2.6. Data Source, Data Processing, and Data Distribution

The RNA-seq data and the corresponding clinical data of overall survival (OS) were downloaded from Gene Expression Omnibus (GEO) (https://www.ncbi.nlm.nih.gov/geo/ data portal. Gene expression profiles of 46 samples with primary ES deposed at GSE63155 (*n* = 46) were used (https://www.ncbi.nlm.nih.gov/geo/query/acc.cgi?acc=GSE63155 (accessed on 2 February 2021)) [21]. Due to the lack of data on relapses, information about the end of the experiment (treatment) for patients with the status of “survivor” was regarded as definitively true. For the analysis, gene expression data from all 46 patients was used without censorship. The normalized data were uploaded by the authors and retrieved from the data portal mentioned above. The LIMMA R package was used to identify differentially expressed genes (DEGs). Gene probe IDs for which an “-/NA” notation was not available were removed. To obtain differential expression profiles, the GEO data were divided into two groups with respect to survivorship (*n* = 32, survivors) and non-survivors (*n* = 14) after receiving treatment.

### 2.7. Functional GO Enrichment Analysis

Relative DEG lists were analyzed by a web server for functional enrichment analysis. g:Profiler Datasets with adjusted *p* < 0.05 (Bonferroni correction) were deemed meaningful enrichment pathways. The “Cluster Profiler” R package was used to combine gene annotation and gene expression analysis results. The results are visualized in a dot-plot.

### 2.8. Experimental Validation of Fold-Change Values Using Semiquantitative RT-PCR Analysis

QIAzol lysis reagent (Qiagen, Germantown, MD, USA) was used to isolate total RNA from 14 ES patients (6 females and 8 males). RNAase-free DNAase1 (Invitrogen, Waltham, MA, USA) was used to remove residual genomic DNA in isolated total RNA. Total RNA was quantified using a Nanodrop spectrophotometer (Beckman Coulter, Inc., Brea, CA USA). Once quantified, 10 ng of total RNA per sample was used to synthesize single-strand cDNA with the iScript cDNA synthesis kit (Bio-Rad, Hercules, CA USA) for one-step RT-PCR analysis. The synthesized single-strand cDNA of 100 and 50 ng per sample was utilized as a template for semi-quantitative PCR analysis with an Encyclo Plus PCR kit (Evrogen, Moscow, Russia) following the manufacturer’s instructions (https://evrogen.com/products/Encyclo-PCR-kit/Encyclo-PCR-kit.shtml (accessed on 2 February 2021). Primer pairs for PCR analyses were selected from previous studies [22]. All PCRs were performed on three replicates per cDNA sample. To obtain the fold change, MGST1 and COL6A2 values of analyzed samples were normalized to β-Actin mRNA expression, as an endogenous reference gene.

### 2.9. Statistical Analyses

GSE63155 includes an analysis of whole-genome expression, whereas GSE146221 is a single-cell analysis of three established ES cell lines (TC71, CHLA9, and CHLA10). The GSE146221 data were generated by 10× Genomics single-cell RNA-sequencing, whereas GSE63155 was generated using Affymetrix platforms. Cox regression analysis was performed for GSE63155 and gene expression correlation analysis for GSE146221. DEGs analysis was performed between groups of survivors and non-survivors, and between individuals with UMAP clusters for GSE63155 and GSE146221 datasets, respectively. Using proteomic profiles of ES36, ES36-DOX, M19, and M19-DOX, we compared DEPs between the following groups: ES36 vs. M19, ES36 vs. ES36-DOX, and M19-DOX vs. ES36-DOX samples, respectively. Our single-cell transcriptomic dataset was used to compare DEGs between clusters 11 and 15 of ES36 vs. M19, ES36 vs. M19 cells and individual UMAP clusters. All analysis was conducted with a statistical significance limit of *p* = 0.05. DEGs analysis of scRNA clusters was performed by the Seurat package R with a statistical significance limit of *p* adjusted = 0.05. All statistical analyses were performed using GraphPad Prism 8.3.0 and R stat Version 4.0.2 (R-project.org (accessed on 25 January, 2022)), and R packages were obtained from the Bioconductor project (www.bioconductor.org (accessed on 25 January 2021)). Statistical significance was set at *p* < 0.05.

### 2.10. Availability of Data and Materials

The datasets (SRA transcriptomic access data: PRJNA846952 and proteomic access data: in progress) used and/or analyzed during the current study are available.

## 3. Results

A significant number of genes are elevated in ES tumor tissue.

To identify DEGs that are relevant to tumor cell survival, we analyzed the GSE63155 microarray database after normalization. Based on the patient survival status (survivors (*n* = 32) vs. non-survivors (*n* = 16)), we applied the univariate and multivariate Cox proportional hazards regression methods for 48 tumor tissues available for analysis (Table 1). 

**Figure 1:**
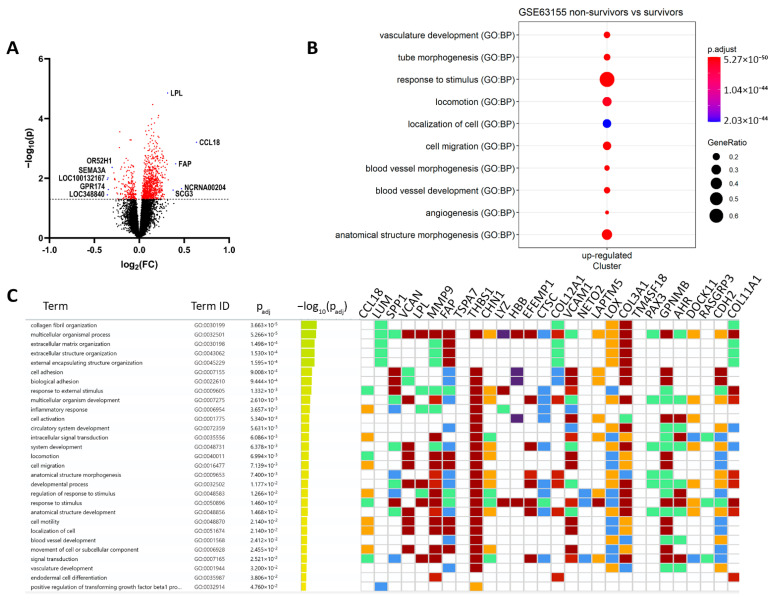
Differentially regulated genes (DEGs) in ES patients with various survival statuses. (**A**) Volcano plot diagram of differentially regulated genes in the tumor tissue from survivor (*n* = 32) vs. nonsurvivor (*n* = 14) patient samples (data were extracted from GSE63155). The data were extracted from GSE63155 and resulted in 1006 genes. Differentially regulated genes with *p* ≤ 0.05 are labeled red. The *x*-axis represents Log2 fold change; the *y*-axis represents negative log10-transformed *p*-values. Dots on the left side represent transcripts upregulated in survivors, and dots on the right side represent transcripts upregulated in non-survivors. Blue dots represent most and downregulated genes by log2fold change. (**B**) a GO analysis of DEGs associated with ES patient survival. The node sizes indicate gene counts enriched in the pathway and the color shows the *p*-value from low (red) to high (blue) level. Significance was set at *p* < 0.05. DEGs, differentially expressed genes; GO, gene ontology. The plot was created using the R gProfiler2. (**C**) Significant enrichment of molecular function of interacting proteins; gene-ontology-based GSEA analysis indicates the top 30 genes most expressed in non-survivor vs. survivor patient samples. The heatmap indicates a log2 fold change in mRNA expression. The colors for different evidence codes in the table: red: inferred from experiment, direct assay, mutant phenotype, genetic interaction, physical interaction; purple: inferred from experiment, direct assay, mutant phenotype, genetic interaction, physical interaction; green: traceable author, non-traceable author, inferred by a curator; orange: expression pattern, sequence or structural similarity, genomic context, sequence model, sequence alignment, sequence ontology, the biological aspect of the ancestor, rapid divergence; blue: reviewed computational analysis, electronic annotation.

Both analyses showed that an event-free survival (EFS) event was a risk factor, while gender, age, primary tumor site, and tumor content did not correlate with OS. Keeping the cutoff value strict among 17,634 genes, genes with *p*-value < 0.05 of their expression between the two groups produced DEGs and the data were depicted in a volcano plot (Figure 1A). Using one-way analysis of variance, we found that multiple transcripts were upregulated in the non-survivor tumor samples. Overall, the Cox regression revealed 812 and 194 genes that were up- or downregulated during the analysis, respectively. Assuming that benefits for ES patient survival occurred from the inhibition of neoplastic signaling inside tumors, we performed GO term analysis. As shown in Figure 1B,C, the GO analysis of upregulated genes revealed an association of DEGs with blood vessel formation, angiogenesis (PECAM1/TGFBI/CD34/THY1/SEMA6A) and cell migration (MMP9/CCL21/MMP2/AKT3/ITGA6/FLT1). At the same time, GO analysis indicates that DEGs mainly regulate cellular metabolism, including cytochrome P450 and glutathione transferase (Table 2). 

To investigate further whether some of these genetic traits are related to clinical prognosis, clinical information was extracted from the GSE63155 database. Based on that information, the top 30 genes overexpressed in the non-survivor group were CCL18, LUM, SPP1, VCAN, LPL, MMP9, FAP, TSPAN7, THBS1, CHN1, LYZ, HBB, EFEMP1, CTSC, COL12A1, VCAM1, C8orf4, NETO2, FAM198B, LAPTM5, LOX, COL3A1, TM4SF18, PAX3, GPNMB, AHR, DOCK11, RASGRP3, CDH2, and COL11A1, mostly responsible for extracellular matrix organization (GO:0030198) and cell adhesion (GO:0007155). Among the abundant genes with high mRNA expression, significantly worse OS was detected for *SPP1* (Hazard Ratio, log-rank 8.3), VCAM1 (7.9), CCL18 (5.1), CTSC (4.6), THBS1 (4.5), and the log-rank p-values were: 7 × 10^−5^, 12 × 10^−5^, 5 × 10^−4^, 1 × 10^−4^ and 1 × 10^−4^ relatively. No statistically significant difference in prognosis was obtained in GEO-deposed patient tumor samples with high and low expressions for LUM (0.056), LYZ (0.18), HBB (0.095), EFEMP1 (0.127), and COL12A1 (0.25) markers. Although previously for ES patients, SPP1 [23], MGST1 [18], and miR34a [24] were considered as markers of ES progression and ES cell sensitivity towards doxorubicin, no additional prognostic factors have been proposed based on individualized treatment options. As chemotherapeutic stress in normal and neoplastic cells results in the production of stress signals, we aimed to establish markers for pathological state and doxorubicin resistance using patient-derived ES cells and embryonic fibroblasts as controls and determine the clinical significance of biomarkers using scRNA-seq and mass-spectroscopy.

### 3.1. Hypoxia and Metastatic Traits Distinguish Tumor Cells from Non-Neoplastic Cells

**Figure 2:**
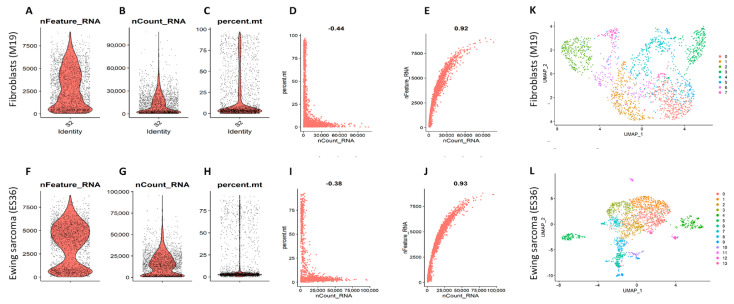
UMAPs of single-cell transcriptome data. Quality control filtering of embryonic fibroblasts (**A**–**E**) and ES36 tumor sample (**F**–**J**)**.** Comparison of 10× Genomics-annotated doublets, singlets, and negatives was assessed using gene expression that was log-transformed using Seurat and presented as violin-box plots with highlighted median values. Violin plots showing the counts of each gene in each cell (**A**,**F**). Violin plots of the sum of the expression levels of all genes in each cell (**B**,**G**). Violin plots of the percentage of mitochondrial genes (**C**,**H**). Scatter plots for the percentage of mitochondrial genes in the sum of the expression levels of all genes in each cell (**D**,**I**). Scatter plots for the counts of genes (**E**,**J).** Embryonic human fibroblasts (**K**) and patient-derived ES (**L**) color-coded by cell identity. Colors show different cell clusters resulting from UMAP clustering using the SEURAT algorithm.

To further investigate the function and significance of differentially expressed genes in the ES cell culture, we conducted a scRNA-seq analysis of ES36 cells (patient-derived ES cells) vs. M19 embryonic fibroblasts. We produced samples’ cDNA libraries using a commercial droplet-based system and sequenced the libraries to obtain transcriptomes covering 500–5000 genes per cell. In total, 5000 cells from each sample were included in the analysis (Figure 2A,L). Overall, we detected an average of 5000 (range 0–10,000) as highly variable genes in the ES36 sample and 5000 (range 0–10,000) in the M19 cells. The GO annotations showed that the functions of genes mainly enriched in tumor cells were related to cell proliferation (GO: 0008283, 37 counts, *p* = 1 × 10^−6^) and cellular adhesion (GO: 0007155, 17 counts, *p* = 7.1× 10^−6^). Inhibited genes were mostly represented by respiratory chain complex I (gamma subunit) mitochondrial (CORUM: 2919, 2 counts, *p* = 0.004) signaling. Our results found that the top 20 highly variable genes in the tumor populations were: CLSPN, CDCA8, CDC20, ISG15, KIF2C, ALPL, STMN1, IFI6, HMGN2, NASP, FABP3, DHRS3, HSPB7, ID3, PDPN, ERRFI1, SRARP, COL8A2, LINC00337, and SERINC2, and in the M19 populations they were: RHBDL2, CDC20, CDCA8, CLSPN, KIF2C, FABP3, ISG15, STMN1, SFN, CDA, DRAXIN, SNHG12, ERRFI1, E2F2, AL590434.1, PDPN, CCDC30, EPHB2, KLF17, and SERINC2. Of note, M19 and ES36 shared some of the mRNA signatures for CLSPN, CDCA8, CDC20, ISG15, KIF2C, STMN, FABP3, PDPN, ERRFI1, and SERINC2 genes. After filtering the sample’s data (Figure 2A through 2E, and 2F through 2J), we applied the Seurat algorithm to further gene clustering. Fibroblasts and ES cell transcriptomes were visualized with a Uniform Manifold Approximation Projection (UMAP) employing ten principal components.

**Figure 3:**
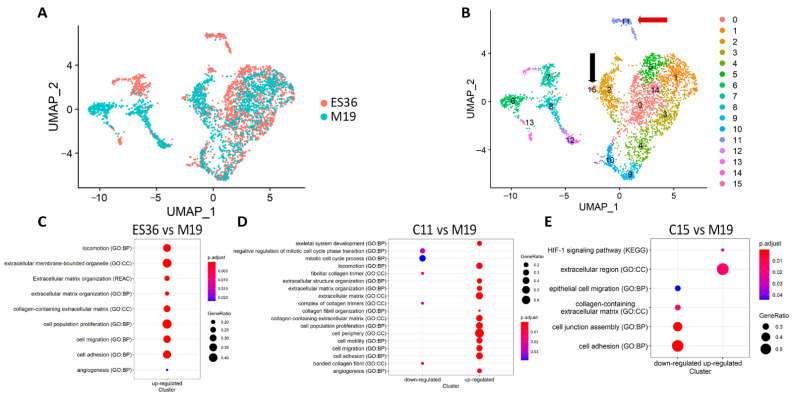
A single-cell ecosystem of embryonic fibroblasts and ES 36 cells. (**A**) Datasets for 10× Genomics analysis were derived from M19 fibroblasts and short-lived patient-derived ES culture (ES36). The 10× Genomics plots identify groups of cells with similar expression patterns. The average of 5000 cultured M19 cells and ES36 cells were green or red color-coded. (**B**) Distribution of single-cell population for each type of cell in the UMAP plot (first two dimensions) in accordance with the clusters made using the Seurat algorithm. Arrows point to the unique clusters that present in ES-patient-derived ES36 tumor culture; GO term analysis that summarizes the biological function and cellular component was performed for DEGs in ES36 tumor cells cluster 11 (*n* = 131 cells) (**C**) or cluster 15 (*n* = 18 cells) (**D**) vs. M19 cells, and also between these ES36 clusters (11 vs. 15), respectively (**E**). The specificity of gene expression value has been normalized and visualized as a circle that corresponds with the ratio of gene expression in ES36 cells vs. M19. The size of nodes indicates the number of cells in each cluster with that ratio.

The multiple sequencing runs from M19 and ES36 cells were combined into a single uniform manifold approximation and projection (UMAP) for visualization (Figure 3A) and clusterization (Figure 3B). Comparable trends of subset colocalization between M19 and ES36 cultured cells remained, reinforcing the conclusion that common gene expression by each cell type is a shared phenomenon regardless of cell type. Across the UMAP plot, we found a distinct number of clusters in which a majority of cells consisted of ES36 (clusters 11, and 15), or mixed (clusters 0, 1, 2, 3, 4, 5, 6, 7, 8, 9, 10, 12, 13, 14, and 15) cells. Furthermore, analysis of C11 and C15 revealed a unique distribution of upregulated genes (Figure 3C,D). Thus, C11 mostly accumulates cells with genes responsible for cell motility, cell adhesion, and migration; in C15, most upregulated genes participate in HIF1 signaling, and downregulated genes are involved in cell adhesion. Overall, in ES cells, MALAT1, FTH1, FTL, VIM, MT-CO1, TMSB4X, FN1, MT-CO2, NEAT1, S100A6, EEF1A1, CALD1, MT-CYB, TPT1, RPL41, TAGLN, RPLP1, LGALS1, TMSB10, CD63, RPS2, ACTB, RPS18, RPS8, RPL37A, RPL13, ANXA2, COL1A2, RPL10, and ACTG1 were most upregulated compared to their expression in M19 cells.

### 3.2. Embryonic Fibroblasts (M19) and ES (ES36) Cells Are Genetically Close

**Figure 4:**
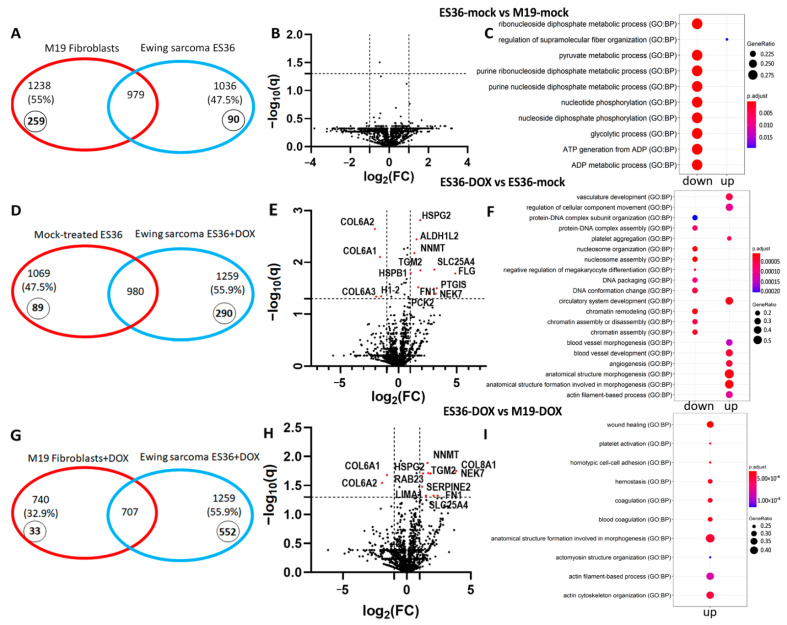
Human fibroblasts and ES cells ES36 display distinct protein compositions. (**A**,**D**,**G**) Venn diagram showing common and unique proteins in mock- or doxorubicin (DOX)-treated M19 and patient-derived ES36 cells. Overlapped proteins were identified in three replicates per each sample. Unique proteins for each type of cell and/or treatment are marked in bold and italics inside small circles. Created using Microsoft Office 2010. (**B**,**E**,**H**) Differential protein expressions between M19 and ES36 samples. Volcano plots of protein abundance between samples showing the distribution of proteins identified in ES36 and M19 during mock or doxorubicin treatment. Data presented as Log2 fold ratio between samples. The most significant genes are highlighted in red and we used an absolute threshold q(FDR) ≤ 0.05 and |log2(FC)| ≥ 1 (**C**,**F**,**I**) Gene ontology (GO) enrichment analysis for 60 potential targets (30 upregulated and 30 downregulated) of ES36 vs. M19, ES36-DOX vs. ES36-mock treated, and ES36-DOX vs.M19-DOX samples with *p* < 0.05 and most value of |log2(FC)| for more complete enrichment. The dot-plot of top-upregulated and downregulated biological functions was performed using *p* adjusted < 0.05. Hierarchical clustering of the protein molecular signature of ES36 and M19 was generated by the computational tool R gProfiler2. Supplemental Tables S1–S3 provide details of all molecular signatures.

To investigate whether transcriptional regulation in ES cells cooperates with protein expression, we performed a proteomic analysis of fibroblasts and ES tumor cells (Figure 4A). Specifically, we analyzed protein expression in mock or DOX-treated M19 and ES36- tumor cells to identify potential signature proteins and pathways that distinguish two types of cells and identify potential targets for future therapeutic interventions. We identified a total of 1069 (47.5%) and 1238 (55%) proteins in ES36 and M19, respectively, out of 2250 possible proteins (Figure 4A,B). For the identification of DEPs, we used a *p*-value ≤ 0.05. This proteome coverage was highly reproducible across the three biological replicates. Proteins that were identified in only one or two biological replicates (1415 (62.9%) of all identified proteins in ES36 and 1454 (64.6%) in M19) were excluded from downstream analysis. Comparative proteomic analysis revealed a subset of 979 proteins common to ES36 (out of 1765) and M19 (out of 1642). A protein signature associated with the cellular amide metabolic process (GO: 0043603) and translation (GO: 0006412) was revealed by the GO term category.

**Figure 5:**
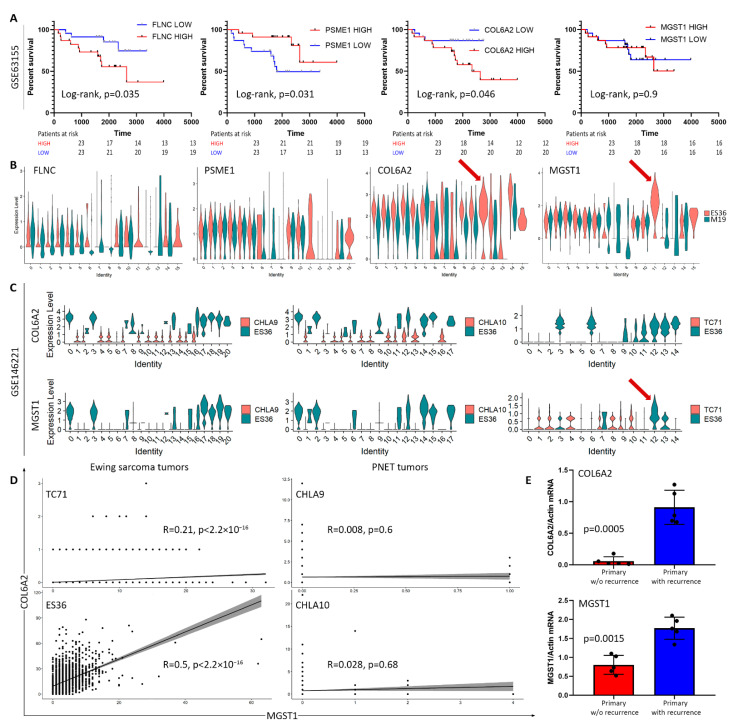
Validation of COL6A2 and MGST1 as markers for ES resistance and progression. (**A**) Group of 46 patients with ES was divided based on their marker expressions. Kaplan–Meier plot of OS for ES patients (GEO datasets GSE63155) stratified by transcriptomic data. The *p*-value was calculated using a two-sided log-rank test. (**B**,**C**) Single-cell RNA sequence discriminates a subset of tumor cells enriched with COL6A2 and MGIST1 markers across subclusters at ES36 and M19 cells (**B**) and in comparison with cellular subsets from TC71, CHLA9, and CHLA10 (**C**) (GEO dataset GSE146221). Data presented as violin plots are based on relative mRNA expression and clusterization of TC71, CHLA9, and CHLA10 with ES36 cells. Blue and black arrows indicate the association of MGST1 and COL6A2 with cluster 11 of ES36 cells. (**D**) Correlations between COL6A2 and MGIST1 expression in various ES cells. A significant positive correlation between COL6A2 and MGIST1 mRNA expression was detected in relapsed ES-based patient-derived cells and no such correlation was seen in PNET was considered significant. (**E**) mRNA levels of MGST1 and COL6A2 in selected primary patient samples (with/without recurrence, based on clinical information available) obtained from Federal State Budgetary Institution National Medical Research Center of Oncology named after N.N. Blokhin of the Ministry of Health of Russia. Semiquantitative PCR analysis of copy numbers of MGST1 and COL6A2 mRNAs obtained from absolute quantification using after normalization to b-actin. An unpaired t-test with Welch’s correction, *p*-value difference between groups is presented.

From a unique protein perspective, M19 and ES36 cells possess 259 and 90 proteins, respectively. Further comparative pathway analysis revealed significant activation of proteins which regulate the enzyme activity (hydrolase, proteolysis, and peptidases) pathways in ES36-cultured cells, while organic acid and carbohydrate metabolism signaling pathways were found to be mostly regulated in M19-cultured cells. In M19 cells, we observed that of 130 proteins, most were involved in cellular catabolism (Figure 5A,D). Remarkably, whereas the extracellular matrix and cytoplasm become locations for the majority of upregulated proteins in ES36 and M19, the downregulated proteins in these types of cells reside mostly in the cytoplasm. Of note, the majority of most regulated proteins (GAPDH, LDHA, PGK1, TPI1, ENO1, ALDOA, GPI, PGAM1, ALDOC) responsible for the purine, glycolytic, pyruvate or ribonucleoside diphosphate metabolism of ES36 cells were inhibited (Figure 4C). For the top upregulated proteins of ES36 cells, R gProfiler2 reported “protein metabolism” (GO: 0051246), “response to stress” (GO: 0006950), and “regulation of cell death” (GO: 0010941) as unique GO term enrichments, and for the most downregulated proteins, the only GO term enrichments were “catabolic process” (GO: 0008104) and “response to growth factor stimulus” (GO: 0090287). At M19, the top 979 proteins with high expression were involved in “program cell death” (GO: 0012501) and “response to stress” (GO: 006950), and with low expression, the GO terms were “translation” (GO: 0006412) and “intracellular transport” (GO: 0046907). Using the analysis approach, we identified several proteins with differential expression between M19 and ES36. All these proteins are of particular interest since their roles in promoting metastases [25,26] and sarcoma progression [27,28] have been demonstrated.

### 3.3. Proteomic Profiling of DOX-Treated ES36 Cells Provides a Unique Signature

By proteome-based MS analysis, the composition of cytoplasmic proteins from fibroblasts and ES cells after doxorubicin treatment was investigated. First, we analyzed protein signatures between DOX-treated ES36 and mock-treated ES36 (Figure 4D,E). Compared with control cells, 980 common and 164 (*p* < 0.05) variable proteins were detected in DOX-treated ES cells. Among the 164 variable proteins in DOX-treated ES36 cells, 107 were upregulated and 57 were downregulated. The GO term analysis for downregulated proteins in the tumor-treated cells with DOX suggests suppression of chromatin remodeling and assembly, and vascular development and angiogenesis process, as top upregulated biological functions, can dictate the behavior of tumor cells in the presence of DOX (Figure 4F). We also looked for proteins that demonstrated a negative or positive trend in the presence of DOX in ES36 cells vs. mock-treated ES36. The top 30 of these DEPs with high expression were ACTG1, ACTC1, FN1, HSPB1, NNMT, GLS, ACTN1, SLC25A3, HTRA1, RTN4, PLS3, TGM2, ATP5F1A, SLC25A6, FHL2, MYH9, CNN2, VDAC1, MDH2, ALDOA, CKAP4, LIMA1, ATP5F1B, VDAC2, FLNA, CLIC4, CNN3, GARS1, and DSTN; and the top 30 with low expression were: H2AC11, H4C1, H2BC12, H1-2, AHNAK, TFRC, HSPA8, ENO1, CFL1, COL6A1, COL1A1, COL6A3, TUBB4B, COL6A2, COL1A2, COTL1, CAPG, FASN, COL3A1, HNRNPM, PCBP1, KHSRP, LRP1, MTHFD1, FLNC, CUTA, HNRNPH3, SLC1A5, SEPTIN9, and RAB5C. The cellular components for several DEPs, such as HSPA8, YWHAZ, CAPG, COTL1, PDCD6IP, ACLY, ARPC5, PYGB, AK1, SLC25A3, CAPNS1, ARHGAP1, ATP6V1E1, TLN1, LAP3, DCTN2, NAGK, and USP14, were extracellular exosomes, with molecular components such as protein-containing complex binding (GO:MF 0044877) and cadherin binding (GO:MF 0045296). The differential expression suggests a decrease in complementary binding mediated by disordered protein domains of neoplastic cells to the other globular domains, including the core binding region of E-cad [29].

We performed comparative proteomics to distinguish the metabolic and functional changes between embryonic fibroblasts and ES ES36 cells during DOX treatment. DOX exposure induces the expression of 707 common proteins in addition to 33 and 552 unique to fibroblasts or tumor cells, respectively (Figure 4G,H). Among 153 DEPs in the DOX-treated ES36 cells vs. M19, 100 proteins were upregulated and 53 were downregulated. The top 30 proteins with the most differences in means were: ACTB, ACTG1, TAGLN, ACTC1, FN1, HBA, TPM1, LDHA, HBB, HSPB1, NNMT, GLS, CSRP1, PLS3, ACTN1, HTRA1, CALU, CNN2, SLC25A3, MYH9, TGM2, FHL2, ANXA2, LIMA1, SLC25A6, MDH2, EEF1G, PEA15, CNN3, and CLIC4, and the top 30 with decreased expression were: AHNAK, TFRC, HSPA8, COL6A1, COL6A3, PDIA6, TUBB4B, KRT1, KRT10, COL6A2, COL1A1, CTTN, NAMPT, PDIA4, YWHAQ, P4HA2, GANAB, PSME1, PSME2, ITGB1, RANGAP1, PRKCSH, SEPTIN9, LRP1, COL3A1, FLNC, CPNE3, PLOD1, ATP1A1, and ANXA11. The GO term analysis for downregulated proteins in ES36 cells treated with DOX showed peptide cross-linking (GO:0018149); in particular, fibrillar collagen trimer (GO:0005583), collagen chain trimerization (REAC: R-HSA-8948216) and focal adhesion (KEGG:04510). The GO term analysis for upregulated proteins was: platelet aggregation (GO:0070527), platelet activation (GO:0030168), homotypic cell–cell adhesion (GO:0034109), actin cytoskeleton organization (GO:0030036), coagulation (GO:0050817), hemostasis (GO:0007599), cytoskeleton organization (GO:0007010), cell adhesion (GO:0007155) (Figure 4I). Of the regulated proteins, eight out of eleven proteins such as SLC25A6, EEF1G, PEA15, TUBB4, SEPTIN9, COL3A1, HBB, and HBA did not have any probes in the GEO dataset (GSE 63155). At the same time, PSME1 expression was associated with the improvement of ES patient survival (Log-rank test, *p* = 0.031), and COL6A2 and FLNC (Figure 5A) showed a negative impact on the survival of ES patients (Log-rank test, *p* = 0.046, and *p* = 0.035 relatively), and involvement in the regulation of integrin and ERK pathways (www.genecards.com (accessed on 1 February 2021)). Of note, the MGST1 marker was detected in two out of three samples in ES36-DOX and three out of three samples in M19-DOX. Although our strict policy on replicates excludes MGST1 from proteomic analysis, we compared COL6A2, PSME1, FLCN, and MGST1 for their clinical significance, correlation with clinical signs, and correlation against each other. We noticed that MGST1 expression was unique to ES36 Cluster 11 (C11), the same cluster where the gene’s subset enriched with COL6A2 (Figure 5B). Furthermore, cluster assignment and overlay of the gene profiles between ES36 and CHLA10 patient-derived ES cells suggest sharing of the small gene population, including MGST1 and COL6A2 genes (Figure 5C). Therefore, mRNA expression of these biomarkers is straightforward in GEO datasets using TC71 and ES36 profiles (ES cells). Using randomly selected ES primary specimens with known clinical history, we analyzed the expression of COL6A2 and MGST1. Validation of scRNA-seq differentially expressed genes (DEGs) through semiquantitative real-time-polymerase chain reaction (RT-PCR) suggests the direct association of COL6A2 and MGST1 mRNAs with ES relapse, demonstrating a direct link between ES36 resistance, tumor regrowth, and increased tumor cell survival due to tumor resistance.

## 4. Discussion

We provide fresh evidence for tumor-cell-signaling activity generated from the interplay of metabolism, stress, and growth pathways that control ES treatment (Figure 4C). Our findings identify key regulatory transcription factors and kinases, as well as novel interactions between these pathways that drive distinct phases of this response in fibroblasts and tumor cells, and link well-characterized signaling mechanisms across cells of the same origin (embryonic fibroblasts and primary short-lived tumor cell cultures of ES36). To avoid the impact of interpatient heterogeneity of the cell signaling, our findings also suggest that it may be necessary to obtain fibroblasts and tumor cells from the same patient with Ewing sarcoma and carefully assess their transcription programs to validate differences in cellular signaling.

The treatment of ES is complicated by heterogeneity both internally and between tumors. To develop an effective anticancer therapeutic, a better understanding of tumor response is necessary. In this regard, reliable biomarkers may represent a source of therapy efficacy against tumor cells. Previous genomic analysis indicates several biomarkers relevant to glutathione metabolism (MGST1) [18] and cell-cycle upregulation mediated by ectopic mir34a [24] overexpression, supporting our GEO analysis of surviving patients with resected tumor samples enriched with cellular metabolism, including cytochrome P450 and glutathione transferase DEGs. Although these studies allow us to explore the intertumoral response to the therapeutic options, mainly chemotherapy, they also utilize responses from normal healthy cells that are present among tumor cell populations. To distinguish cells, we compared the first basal level of DEG expression between patient-derived ES36 and M19 embryonic fibroblasts and then we clustered the single-cell transcriptome which allowed us to detect 14 and 8 clusters, respectively. As a result of M19 and ES transcriptomics mixing, we detected a few unique clusters in ES36 cells (C11 and C15). Using the GO term biological functions, a member of a gene family that is relevant to migration was associated with the C11 cluster. Among genes, the highest expression detected in C11 and C15 of ES cells were: FTL, SERPINE2, CCDC80, CALD, and COL1A2 or FTL, TIMP1, TAGLN, SH3BGRL3, and CALD1 relatively. Despite the involvement of migration for the hub of eight genes, only COL1A2 was previously related to the OS of patients with ES [30]. On the molecular level, a reduced level of COL1A2 was inversely correlated with osteosarcoma proliferation and migration under cisplatin restriction [31]. Besides COL1A2, the ferritin light chain (FTL) expression was shown in association with the response to chemotherapy [32] and contributed to the proliferation, migration, and invasion [33] of osteosarcoma cells. SERPINE2 may drive self-proliferation and drug resistance in osteosarcoma [34], and has poor prognosis for patients with bladder [35] and ovarian [36] cancer with high COL1A2 expressions.

It is noteworthy that in the case of ES36 DOX vs. ES36 MOCK, we found a significant decrease in the expression of all three alpha-helices of the COL6 protein (Figure 4E), including COL6A2. This effect means a more complex effect of doxorubicin therapy on ES36, mainly, changing the content and 3D structure of the extracellular collagen matrix. At the same time, the expression of COL6A1 and COL6A2 is also significantly reduced in ES36 DOX versus M19 DOX (Figure 4H), which also indicates a specialized and unique functional nature of the decreasing COL6 level in ES36 cells in response to DOX. This may indicate the involvement of COL6 in the formation of vulnerability to doxorubicin, similar to breast cancer cells [37]. Whether the expression of MGST1 alone or in combination with COL6A2 is required for metastatic development has yet to be seen. However, our working hypothesis is that ES metastatic development might require multiple gene activation depending on the tumor stage. For instance, whereas MSGT1 and COL6A2 expression is required for ES cell survival during chemotherapeutic stress mediated by Doxorubicin, SOX2, KL4 and/or Oct4 [38] are vital for maintaining the stemness of ES cells and for the ES seeding and survival, as second-nodule activation of ZEB2 [39] and IGF-1R [40] transcriptional programs are most helpful. Among those, at some points of ES, such as seeding and cells making decisions to colonize bone or lung, additional proteins become influential, such as neuropeptide Y [41] or Caveolin-1 [42]. However, some studies [43] demonstrated the involvement of ES-based cellular proteins in the ability of ES to metastasize in vitro; the ultimate test for metastatic progression has required the presence of a microenvironment to support the invasion, migration, internalization and seeding of ES tumor cells. Therefore, in vivo tail injection [42,44] or injection into a muscle such as a calf [45] or gastrocnemius muscle [41,46] with tumor cells, or the use of the metastatic Ewing sarcoma mouse model [47] may hold the answers on ES seeding and metastasis development.

DOX-mediated stress is a complex cellular reaction produced by sarcoma cells. It is unclear why tumor cells express two proteins, one of each encoded alpha chain of collagen type VI and the second a protein with high glutathione transferase activity. It is generally agreed that MGST1 possesses a protein homodimerization activity, suggesting that stress response employs the MGST1 expression signal as a stressor signal for collagen filament re-engagement. It is also plausible that COL6A2 serves as an adapt messenger function to unify the cellular response during DOX stress and disrupt cellular adaptation via cooperation with other proteins. A possible degree of cooperation has been demonstrated previously for DSCAM and COL6A2 in the H9C2 cardiac cell line. In these cells, the transcriptional analysis of that interaction points to genes involved in adhesion and cardiac hypertrophy [48] that cause severe physiological and morphological defects in the heart of Drosophila. Further evidence suggests that glutathione and glutathione-related enzymes are at the forefront of the adaptive detoxification cellular response [49] to stress during DOX-mediated ROS production. Considering that DOX-resistant cervical cancer cells also display activation of GSH signaling, including MGST1 gene expression isoforms [50], activation of such proteins might represent a DOX-specific stress network that elaborates cellular function during the stress and becomes a common feature for various tumor cells. Crosstalk between various cellular functions mediated by COL6A2 and MGST1 coordinates tumor cell vulnerability and might define the severity of the ES cells’ damage.

## 5. Conclusions

Based on the results of our study, by clustering scRNA-seq and subsequent DEG analysis, we were able to perform genotypic profiling of ES cells. Further proteomic and DEGs of GSE63155 analysis identified and showed the main differences in the expression of metastatic and primary-tumor markers that can be considered for targeted chemotherapy. Our data suggest that doxorubicin treatment mediates the induction of collagen remodeling and activation of glutathione metabolism, which allows premetastatic phenotype changes in ES tumor cells.

## Figures and Tables

**Table 1:** Univariate and multivariate Cox analysis of the correlation clinical factors with OS in patients with ES.

	Univariate			Multivariate		
	Beta	HR (95% CI)	*p*.Value	Beta	HR (95% CI)	*p*.Value
Sex	−0.47	0.62 (0.21–1.9)	0.4	−2.358	0.095 (0.005–1.84)	0.120
Age_at_enrollment_days	0.00033	1 (1–1)	0.07	−0.004	0.996 (0.98–1.0)	0.465
Age	0.11	1.1 (0.99–1.3)	0.075	1.637	5.14 (0.085–309.385)	0.433
Primary_tumor_site	−0.28	0.76 (0.35–1.6)	0.47	1.502	4.492 (0.377–53.53)	0.235
Efs_event	2	7.7 (3.4–17)	9.5e-07	3.941	51.476 (3.736–709.32)	0.003
Tumor_content	0.77	2.2 (0.57–8.2)	0.26	−0.562	0.57 (0.088–3.676)	0.554

Abbreviations: ES, Ewing sarcoma; EFS event; HR, hazard ratio; 95% CI, 95% confidence interval.

**Table 2:** The biological process and pathways regulated by DEGs.

	Description	ID	*p* Value	Count/Gene ID
Downregulated	locomotion	GO:0040011	1.14 × 10^−26^	CCL18/VCAN/MMP9/FAP/THBS1/CHN1/VCAM1/LOX/COL3A1/GPNMB/CDH2/SULF1/GJA1/ACTA2/TGFBR2/SMOC2/CCL21/MMP2/DCLK1/AKT3/CD200/MAP1B/RND3/KDR/MCC/LEF1/SGK1/SDC2/ENPP2/PECAM1/KITLG/CCR1/ADAMTS1/ITGA6/EPS8/ADAMTS9/EFNB2/CD34/THY1/SEMA6A/LYN/ALX1/S100A8/ITGA9/MTUS1/NRP1/PLVAP/PRKD1/ETS1/LYVE1/CDH5/PLXNC1/PODXL/ITGA2/FCER1G/TLR4/TEK/NRP2/PDGFRB/LAMB1/ELMO1/EMP2/MCTP1/FLT1/DOCK2/PTPRC/XBP1/CHL1/SATB2/PLXNA2/DLC1
	angiogenesis	GO:0001525	4.39 × 10^−26^	FAP/THBS1/GPNMB/SULF1/THBS2/PTPRB/TGFBR2/SMOC2/MMP2/CALCRL/CYBB/ADAM12/AKT3/ANPEP/KDR/LEF1/ENPP2/COL15A1/CEMIP2/TGFBI/APLNR/ADAMTS1/ADAMTS9/EFNB2/CD34/THY1/SEMA6A/SAT1/NRP1/PRKD1/ETS1/COL4A1/CDH5/EMCN/EPAS1/TEK/COL4A2/NRP2/PDGFRB/EMP2/FLT1/XBP1
	positive regulation of cell migration	GO:0030335	2.52 × 10^−19^	MMP9/THBS1/GPNMB/ACTA2/TGFBR2/SMOC2/CCL21/MMP2/AKT3/KDR/LEF1/ENPP2/PECAM1/KITLG/CCR1/ADAMTS1/ITGA6/THY1/SEMA6A/LYN/NRP1/PLVAP/PRKD1/ETS1/LYVE1/CDH5/PODXL/ITGA2/TLR4/TEK/NRP2/PDGFRB/LAMB1/FLT1/PTPRC/XBP1
	cell differentiation	GO:0030154	6.24 × 10^−16^	SPP1/VCAN/LPL/MMP9/CHN1/EFEMP1/COL12A1/VCAM1/LOX/COL3A1/DOCK11/CDH2/COL11A1/KRT10/SULF1/STEAP4/GJA1/A2M/ACTA2/TGFBR2/CCL21/MMP2/CD53/GPM6B/DCLK1/ADAM12/PLEK/MAP1B/ANPEP/KDR/ESRP1/LEF1/SGK1/SDC2/PECAM1/COL15A1/KITLG/CCR1/TGFBI/APLNR/ITGA6/MAP2/ADAMTS9/MTSS1/EFNB2/CD34/CD36/THY1/SEMA6A/LYN/HEY2/RAI14/ALX1/S100A8/RPS6KA2/SPRY4/NRP1/PRKD1/TMEM119/ETS1/PRICKLE1/COL4A1/CDH5/PLXNC1/PODXL/ITGA2/SLC6A6/EPAS1/FCER1G/TLR4/TEK/COL4A2/FRMD6/NRP2/PDGFRB/LAMB1/EMP2/FLT1/FARP1/DOCK2/PTPRC/XBP1/CHL1/RPS6KA3/SATB2/PLXNA2/TAGLN/BHLHE40
	extracellular matrix organization	GO:0030198	8.07 × 10^−14^	LUM/MMP9/FAP/COL12A1/LOX/COL3A1/COL11A1/SULF1/SMOC2/MMP2/GPM6B/COL14A1/MMP16/COL15A1/TGFBI/ADAMTS1/ITGA6/ADAMTS9/ITGA9/FBLN5/COL4A1/ITGA2/COL4A2/LAMB1/NID2
	cell-substrate adhesion	GO:0031589	7.69 × 10^−11^	THBS1/VCAM1/COL3A1/EDIL3/CCL21/GPM6B/VWF/KDR/ITGA6/ADAMTS9/CD34/CD36/THY1/SPRY4/NRP1/FBLN5/LYVE1/ITGA2/TEK/LAMB1/EMP2/NID2/DLC1
	chemotaxis	GO:0006935	7.74 × 10^−11^	CCL18/THBS1/CHN1/VCAM1/LOX/GPNMB/SMOC2/CCL21/KDR/LEF1/ENPP2/CCR1/EFNB2/SEMA6A/LYN/S100A8/ITGA9/MTUS1/NRP1/PRKD1/PLXNC1/ITGA2/FCER1G/NRP2/PDGFRB/FLT1/DOCK2/CHL1/PLXNA2
	cell-matrix adhesion	GO:0007160	1.55 × 10^−9^	THBS1/VCAM1/COL3A1/CCL21/GPM6B/KDR/ADAMTS9/CD34/CD36/THY1/NRP1/FBLN5/LYVE1/ITGA2/TEK/EMP2/NID2/DLC1
	cell-substrate junction assembly	GO:0007044	0.0002	THBS1/GPM6B/KDR/ITGA6/THY1/NRP1/ITGA2/TEK/DLC1
	collagen-containing extracellular matrix	GO:0062023	3.43 × 10^−21^	LUM/VCAN/MMP9/THBS1/EFEMP1/COL12A1/CTSC/COL3A1/CDH2/COL11A1/EDIL3/SULF1/A2M/THBS2/SMOC2/MMP2/VWF/COL14A1/SDC2/SPON1/PCOLCE/COL15A1/TGFBI/ADAMTS1/SPARCL/ADAMTS9/S100A8/BGN/HAPLN1/FBLN5/COL4A1/COL4A2/LAMB1/NID2
	extracellular exosome	GO:0070062	6.20 × 10^−10^	LUM/SPP1/MMP9/THBS1/LYZ/HBB/EFEMP1/COL12A1/CTSC/VCAM1/PPIC/EDIL3/KRT10/STEAP4/A2M/ACTA2/CD53/VWF/ANPEP/MAN1A1/PECAM1/PCOLCE/COL15A1/CEMIP2/TGFBI/EPS8/PRSS23/THY1/LYN/S100A8/CD14/BGN/PLVAP/MYO1B/ENTPD1/FBLN5/LYVE1/RFTN1/PODXL/NT5E/FCGR3A/COL4A2/LAMB1/DOCK2/RAB27B/PTPRC/MARCKS/CHL1/NID2/TNFSF10/PYGL
	collagen trimer	GO:0005581	1.90 × 10^−6^	LUM/COL12A1/LOX/COL3A1/COL11A1/COL14A1/COL15A1/CD36/COL4A1/COL4A2
	collagen binding	GO:0005518	1.53 × 10^−8^	LUM/MMP9/THBS1/LOX/VWF/COL14A1/PCOLCE/TGFBI/SPARCL1/ITGA2/NID2
	glycosaminoglycan binding	GO:0005539	3.92 × 10^−8^	VCAN/LPL/THBS1/GPNMB/COL11A1/SULF1/THBS2/TGFBR2/SMOC2/PCOLCE/ADAMTS1/NRP1/BGN/HAPLN1/LYVE1/NRP2/PTPRC
	Degradation of the extracellular matrix	REAC:R-HSA-1474228	6.00 × 10^−8^	SPP1/MMP9/COL12A1/COL3A1/COL11A1/A2M/MMP2/COL14A1/MMP16/COL15A1/ADAMTS1/ADAMTS9/COL4A1/COL4A2/LAMB1
	Collagen formation	REAC:R-HSA-1474290	4.10 × 10^−6^	MMP9/COL12A1/LOX/COL3A1/COL11A1/COL14A1/PCOLCE/COL15A1/ITGA6/COL4A1/COL4A2
	Collagen degradation	REAC:R-HSA-1442490	2.99 × 10^−5^	MMP9/COL12A1/COL3A1/COL11A1/MMP2/COL14A1/COL15A1/COL4A1/COL4A2
	Collagen chain trimerization	REAC:R-HSA-8948216	0.0004	COL12A1/COL3A1/COL11A1/COL14A1/COL15A1/COL4A1/COL4A2

## Data Availability

The RNA-seq datasets presented in this study can be found in the online repository (SRA transcriptomic access data: PRJNA846952). The names of the repository/repositories used in the study and their accession number(s) can be found below: https://www.ncbi.nlm.nih.gov/geo/, GSE63155, and GSE146221. While we are working to submit the proteomic files to the online repository, please use the following link to download the data (https://kapitul.ru/owncloud/index.php/s/UwSbISThjNXQyWP, password sarcoma); alternatively, data are available upon sending a request to Dr. Ilya Ulasov, ulasov75@yahoo.com.

## Supplementary Materials

The following supporting information can be downloaded at: https://www.mdpi.com/article/10.3390/cancers14225498/s1, Figure S1: Transcriptomic comparison of ES36 versus TC71, CHLA9, and CHLA10 patient-derived ES cells. Supplemental Tables S1–S3: Go terms of DEG expression in ES36 vs M19 fibroblasts, ES36-DOX vs ES36 primary tumor cells, and ES36-DOX (Doxorubicin-treated Ewing’s sarcoma cells ES36) vs M19-DOX doxorubicin-treated human fibroblasts M19
