# Supplementary material for: Identification of Factors Driving Doxorubicin-Resistant Ewing Tumor Cells to Survival
Source: Cancers (Basel). 2022 Nov 9;14(22):5498. doi: 10.3390/cancers14225498 (PMC9688843; doi:10.3390/cancers14225498)
Supplement: Supplementary file 1 [file cancers-14-05498-s001.zip › Table S1.pdf]

Supplemental Table S1. Go terms of DEG expression in ES36 vs M19 fibroblasts.

	Description	ID	P adj value	Count/Gene ID
DOWNREGU	regulation of supramolecular fiber organization	GO:1902903	0,017968	6
UPREGULATED	glycolytic process	GO:00060 96	2,96E-12	9
	ATP generation from ADP	GO:00067 57	3,31E-12	9
	ADP metabolic process	GO:00460 31	7,85E-12	9
	nucleoside diphosphate phosphorylation	GO:000616 5	2,07E-11	9
	nucleotide phosphorylation	GO:004693 9	2,27E-11	9
	purine nucleoside diphosphate metabolic process	GO:000913 5	2,72E-11	9
	purine ribonucleoside diphosphate metabolic process	GO:000917 9	2,72E-11	9
	pyruvate metabolic process	GO:000609 0	3,24E-11	9

	ribonucleoside diphosphate metabolic process	GO:0009185	4,57E-11	9

Abbreviations: DEG, differentially expressed genes; GO, gene ontology;
