# Supplementary material for: Identification of Factors Driving Doxorubicin-Resistant Ewing Tumor Cells to Survival
Source: Cancers (Basel). 2022 Nov 9;14(22):5498. doi: 10.3390/cancers14225498 (PMC9688843; doi:10.3390/cancers14225498)
Supplement: Supplementary file 1 [file cancers-14-05498-s001.zip › Table S2.pdf]

Supplemental Table S2. Go terms of DEG expression in ES36-DOX vs ES36 primary tumor cells.

	Description	ID	P adj value	Count/Gene ID
DOWNREGULATED	nucleosome assembly	GO:0006334	8,02E-07	7
	nucleosome organization	GO:0034728	5,86E-06	7
	chromatin remodeling	GO:0006338	7,53E-06	8
	chromatin assembly	GO:0031497	1,82E-05	7
	negative regulation of megakaryocyte differentiation	GO:0045653	2,29E-05	4
	DNA conformation change	GO:0071103	4,31E-05	8
	chromatin assembly or disassembly	GO:0006333	4,49E-05	7
	protein-DNA complex assembly	GO:0065004	6,36E-05	7

	DNA packaging	GO:0006323	9,11E-05	7
	protein-DNA complex subunit organization	GO:0071824	0,000201	7
UPREGULATED	anatomical structure formation involved in morphogenesis	GO:0048646	5,11E-09	15
	anatomical structure morphogenesis	GO:0009653	1,22E-05	17
	circulatory system development	GO:0072359	1,43E-05	12
	blood vessel development	GO:0001568	2,87E-05	10
	angiogenesis	GO:0001525	3,42E-05	9
	vasculature development	GO:0001944	4,35E-05	10
	platelet aggregation	GO:0070527	6,08E-05	5
	regulation of cellular component movement	GO:0051270	0,0001	11

	actin filament-based process	GO:0030029	0,000111	10
	blood vessel morphogenesis	GO:0048514	0,000142	9

Abbreviations: DEG, differentially expressed genes; GO, gene ontology;
