# Supplementary material for: Identification of Factors Driving Doxorubicin-Resistant Ewing Tumor Cells to Survival
Source: Cancers (Basel). 2022 Nov 9;14(22):5498. doi: 10.3390/cancers14225498 (PMC9688843; doi:10.3390/cancers14225498)
Supplement: Supplementary file 1 [file cancers-14-05498-s001.zip › Table S3.pdf]

Supplemental Table S3. Go terms of DEG expression in ES36-DOX (Doxorubicin-treated Ewing's sarcoma cells ES36) vs M19-DOX doxorubicin-treated human fibroblasts M19.

	Description	ID	P adj value	Count/Gene ID
UPREGULATED	platelet aggregation	GO:0070527	7,09E-09	7
	homotypic cell-cell adhesion	GO:0034109	5,35E-08	7
	platelet activation	GO:0030168	6,19E-07	7
	anatomical structure formation involved in morphogenesis	GO:0048646	1,46E-06	13
	actin cytoskeleton organization	GO:0030036	2,17E-06	11
	wound healing	GO:0042060	5,25E-06	9
	actin filament-based process	GO:0030029	7,44E-06	11
	actomyosin structure organization	GO:0031032	1,16E-05	7
	blood coagulation	GO:0007596	3,24E-05	7
	coagulation	GO:0050817	3,77E-05	7

Abbreviations: DEG, differentially expressed genes; GO, gene ontology;
