# Supplementary figures and images for: Identification of Factors Driving Doxorubicin-Resistant Ewing Tumor Cells to Survival
Source: Cancers (Basel). 2022 Nov 9;14(22):5498. doi: 10.3390/cancers14225498 (PMC9688843; doi:10.3390/cancers14225498)

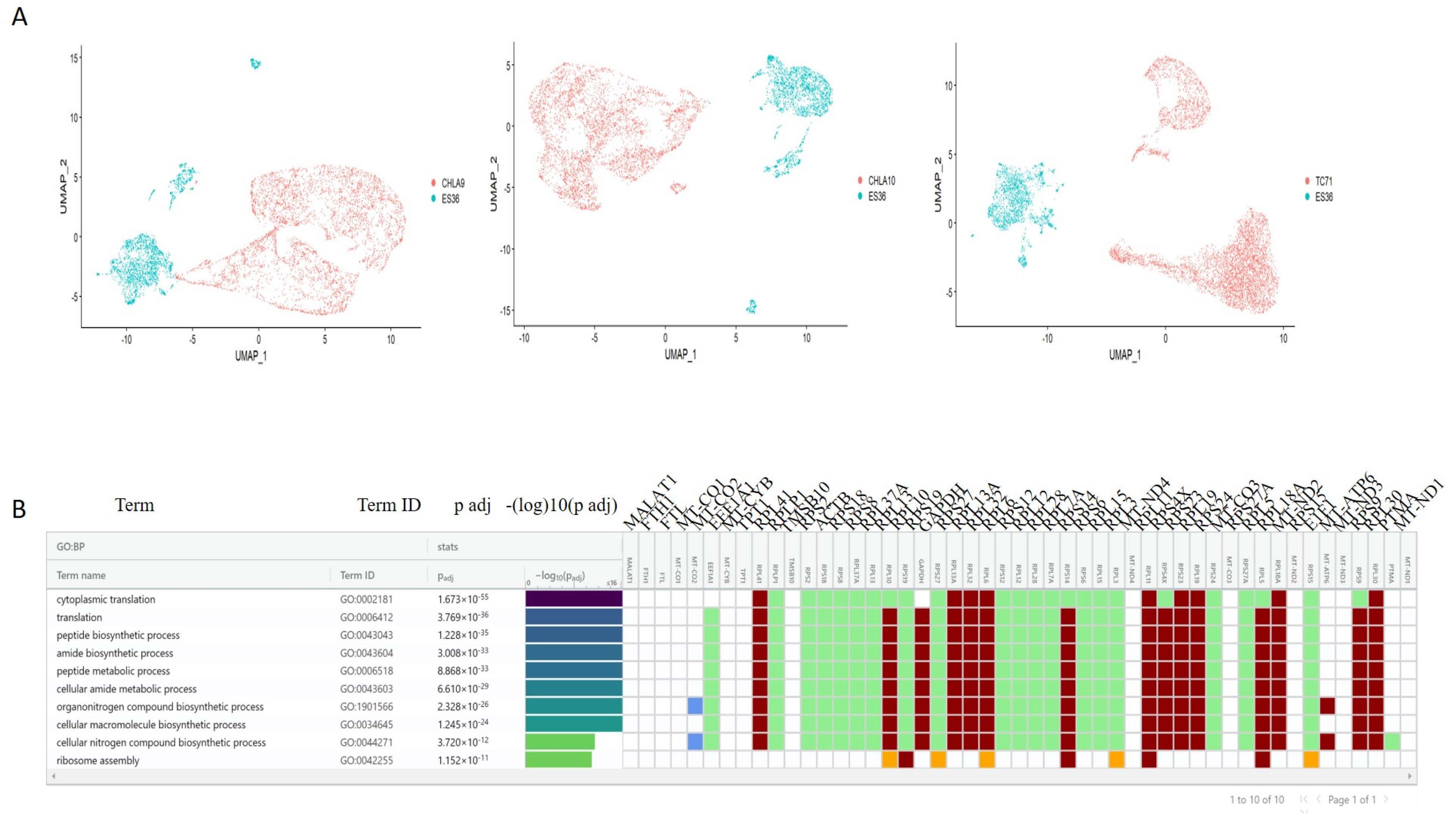

Supplement: Supplementary file 1 [file cancers-14-05498-s001.zip › Figure S1.jpg]
